# Troubling disease syndrome in endangered live Patagonian huemul deer (*Hippocamelus bisulcus*) from the Protected Park Shoonem: unusually high prevalence of osteopathology

**DOI:** 10.1186/s13104-017-3052-4

**Published:** 2017-12-16

**Authors:** Werner T. Flueck, Jo Anne M. Smith-Flueck

**Affiliations:** 1 Swiss Tropical and Public Health Institute, University Basel, Socinstrasse 57, Basel, 4051 Switzerland; 2 National Council of Scientific and Technological Research (CONICET), Buenos Aires, Argentine National Park Administration, C.C. 592, 8400 Bariloche, Argentina; 3 Laboratorio de Teriogenología “Dr. Héctor H. Morello”, Facultad de Ciencias Agrarias, Univ. Nac. Comahue, Cinco Saltos, DeerLab, 8400 Bariloche, Argentina

**Keywords:** Huemul, Hippocamelus bisulcus, Trace mineral deficiency, Selenium, Iodine, Osteopathology, Acute periodontitis, Parodontitis, Lack of recovery

## Abstract

**Objective:**

The last 1500 endangered Patagonian huemul deer (*Hippocamelus bisulcus*) exist in > 100 groups which are not recovering. Prevalence of osteopathology in dead huemul was 57+% (Argentina), whereas similar cases in Chile were accompanied by selenium deficiency. The first clinical cases from live wild huemul confirm widespread osteopathology which explains short life spans, low recruitment, and thus absence of population recovery.

**Results:**

The first-ever radio-collaring of 3 male huemul in Argentina and 3 females, plus a fresh female carcass allowed examination of 7 huemul. Of these, 86% were diseased and clinical pathophysiognomy included lameness, affected hoof, exfoliation of 2–7 incisors, other cranial osteopathologies, and muscle atrophy. The parsimonious explanation for absent population recovery is high prevalence of osteopathology as evidenced earlier in carcasses, and now by these clinical cases. Areas currently used by huemul have reduced selenium bioavailability, very deficient soil levels, and overt selenium deficiency in local livestock and plants. These areas are known to result in primary iodine deficiency which is aggravated by selenium deficiency. The nexus to nutritional ecology of huemul likely is inaccessibility to most fertile lowlands and traditional winter ranges, elimination of migratory traditions, and concomitant elimination of source populations.

**Electronic supplementary material:**

The online version of this article (10.1186/s13104-017-3052-4) contains supplementary material, which is available to authorized users.

## Introduction

Patagonian huemul deer (*Hippocamelus bisulcus*) have been considered endangered for over a century [1], numbers continue declining, and possibly only 1500 individuals remain. Numbers and occupied areas likely began to decline with pre-Columbian humans, and earliest accounts about huemul from interior Patagonia described landscapes already modified for several 100 years [1–3]. In Argentina, only 350–500 huemul remain, split into some 50 subpopulations, and knowledge is rudimentary [4], as tools like telemetry have only recently been employed. Skeletal remains collected between 1993–2007 provided data on bone disease and demonstrated its potential to contribute to morbidity [2]: osteopathy among adults was at least 57%, with affected individuals having mandibular (63%), maxillary (100%), and appendicular lesions (78%). These lesions would affect predator avoidance, possibly explaining the low average adult age (3.1 years) and lack of population recovery. Primary etiologic factors discarded were senescence, gender, fulminating infections, congenital anomalies, parasitism or marasmus, fluorosis, and disorders of metabolic, endocrine, genetic, or neurologic origins. Instead, secondary chronic alveolar osteomyelitis and osteoarthritis were hypothesized to relate to nutritional ecology of huemul. Selenium (Se) deficiency, which impairs bone metabolism and causes periodontitis in ruminants, occurs regionally and is more prevalent at high altitudes where extant huemul tend to remain. This was recently corroborated in Chilean huemul, which were also afflicted with such osteopathology, and severe Se deficiency [5, 6]. The presented novel data about types and prevalence of osteopathology in live huemul extends previously documented postmortem cases [2, 6] and furthermore lends imperative support for the hitherto hypothesized causal relationship between nutritional constraints as factors which have prevented the recovery of most of the > 100 fragmented subpopulations.

## Main text

### Methods

The study area (44°51′S, 71°48′W) has two lakes (167 km^2^) surrounded by the Andes mountains, peaking 1900 m or higher (Additional file 1: Figure S1). Within the subantarctic province it is characterized by mature and dense forests of principally deciduous lenga trees (*Nothofagus pumilio*), which occur from lake level (930 m) up to about 1300 m. Mean winter temperature (Jun–Aug) is − 4 to − 2 °C with mean precipitation between 300–400 mm, principally as snow, and annual precipitation averaging 1000 mm (Additional file 2: Figure S2) [7].

During winter 2017, deer were immobilized by darting, using medetomidine and ketamin reversed by atipamezole. As logistic circumstances were complex (remoteness, deep snow, animals using shoreline to move about), which promoted elevated capture stress, manipulations (morphological measurements, brief gross examinations, photographic documentation, radio-collar installations) were minimal to reduce the down time. Additionally, a female found soon after death was necropsied. Fat depots were measured, bone marrow fat was determined via dehydration, and the skeleton was cleaned for examination [8, 9].

### Results

#### Case 1

A male was noted to have an antalgic gait favoring the right front leg (limping). The coronet diameter of the velvet antler was 3 cm and age judged to be 2.5 years old. No other lesions were apparent and a radio-collar was placed. The antler grew 10 cm within 26 days, but exhibited asymmetrical growth, typical in presence of injuries.

#### Case 2

**Fig. 1:**
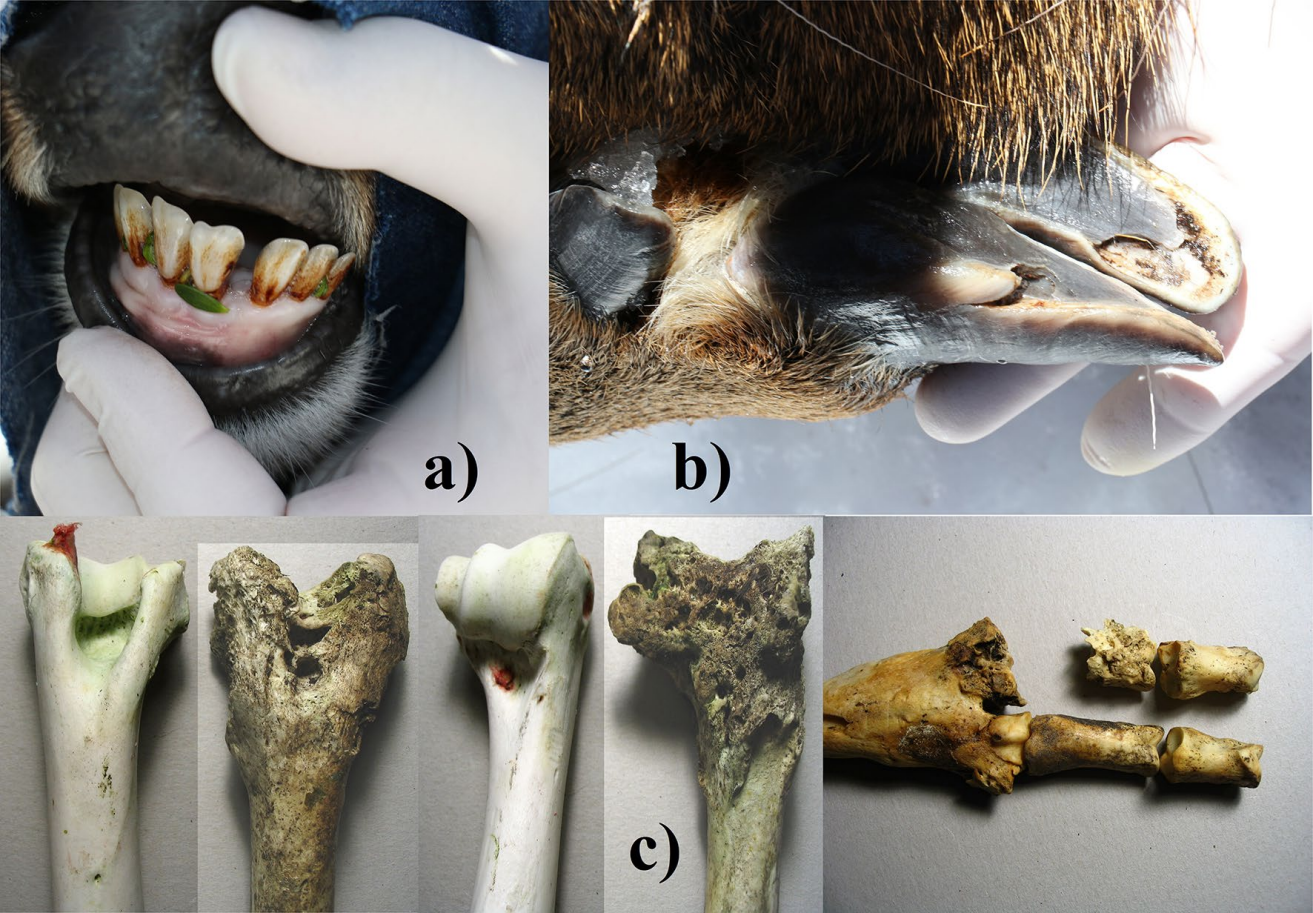
Case 2, male 3.5 years old: **a** receded gum with exposed roots of the central 4 incisors, wide gap between front teeth, and discoloring of the enamel; **b** extensive rip on the abaxial side of the left hoof wall, and partially detached and missing subunguinus (sole); **c** examples of articular lesions from dead huemul of this subpopulation, including foot and humerus [2]

A male with 17 cm long velvet was marked with a radio-collar. The age was 3.5 years old, based on incisor lengths and 5.7 cm of coronet diameter. Wide gaps of 4–5 mm occurred between incisors I1 and I2, gum line recession partially exposed roots of other 4 incisors, and enamel had brownish discoloring (Fig. 1a). An appendicular lesion on the left front hoof included a 3 cm long tear on the abaxial wall, reaching under the frontal third of the callus pad. Moreover, the subunguinus was partially detached and missing (Fig. 1b). These lesions may promote secondary infections, but certainly inflammation and necrosis (Fig. 1c).

#### Case 3

**Fig. 2:**
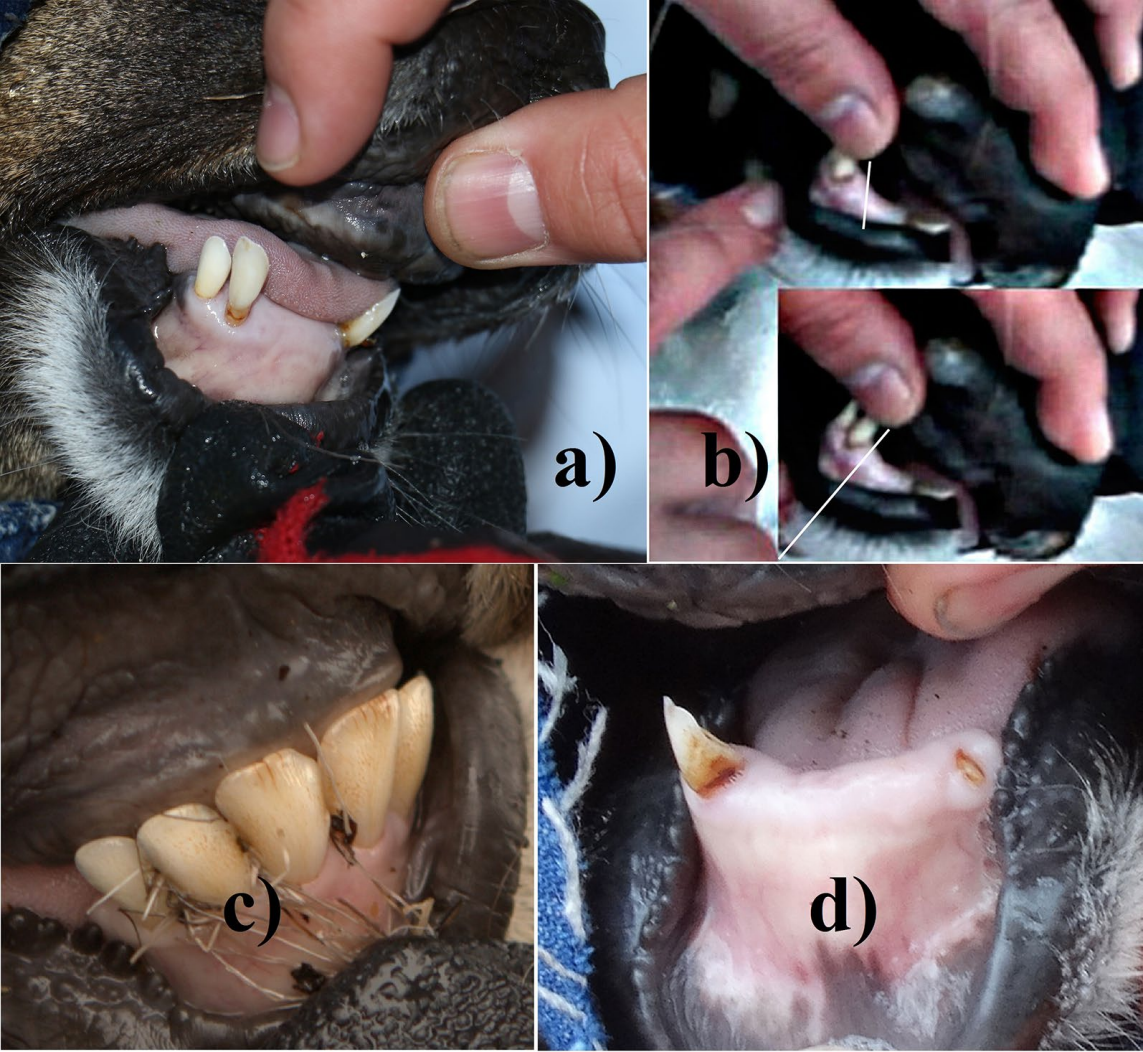
**a** Case 3, female 3.5 years old: loss of 4 central incisors with gums healed over, receded gum and exposed roots of remaining teeth; **b** remaining teeth where completely lose with tips moving about 10 mm; **c** Case 4, female 1.5 years old: damaged front teeth showing pitting and brown staining, incisors with several vertical fissures 2–3 mm in length, and absent canines; **d** Case 5, male 4–5 years old: only 1 front tooth remains; the right canine is broken and only part of the root of the left canine remains at gum level

A female 3.5 years of age, based on incisor length, was marked with radio-collar. She had lost 4 central incisors with gums completely healed, but receded to expose the roots of remaining teeth (Fig. 2a), which were completely loose with tips moving some 10 mm (Fig. 2b). Also, submandibular lymph nodes were swollen. This female was not lactating, palpation revealed a fetus, and she was accompanied by a yearling daughter.

#### Case 4

Based on incisors and behavioral interaction with another mother, a yearling female was marked with radio-collar. The front teeth exhibited pitting and brown staining, central incisors had several vertical fissures 2–3 mm in length, and absent canines (Fig. 2c).

#### Case 5

A male 4–5 years old, based on 7 cm coronet diameter and 23 cm antler velvet length, was marked with radio-collar. Palpation of the spine revealed a progressive stage of muscle atrophy, and according to body condition scoring sources, he qualifies as near-emaciated. The proximate cause undoubtedly related to having lost 7 of 8 front teeth. The remaining canine was broken, only the root of the other canine remained, with the gum in-between completely healed (Fig. 2d).

#### Case 6

A female was crushed by a tree and found soon after based on lack of putrefaction, autolysis and odor. Age was 2.5 years old based on incisor length and tooth wear [10]. Winter conditions helped carcass preservation, but scavengers quickly had removed all entrails, likely because the impact had opened the venter. Several fat deposits indicate low reserves: 5 mm brisket fat, 0 mm rump fat, and 74.3% of femur marrow fat.

**Fig. 3:**
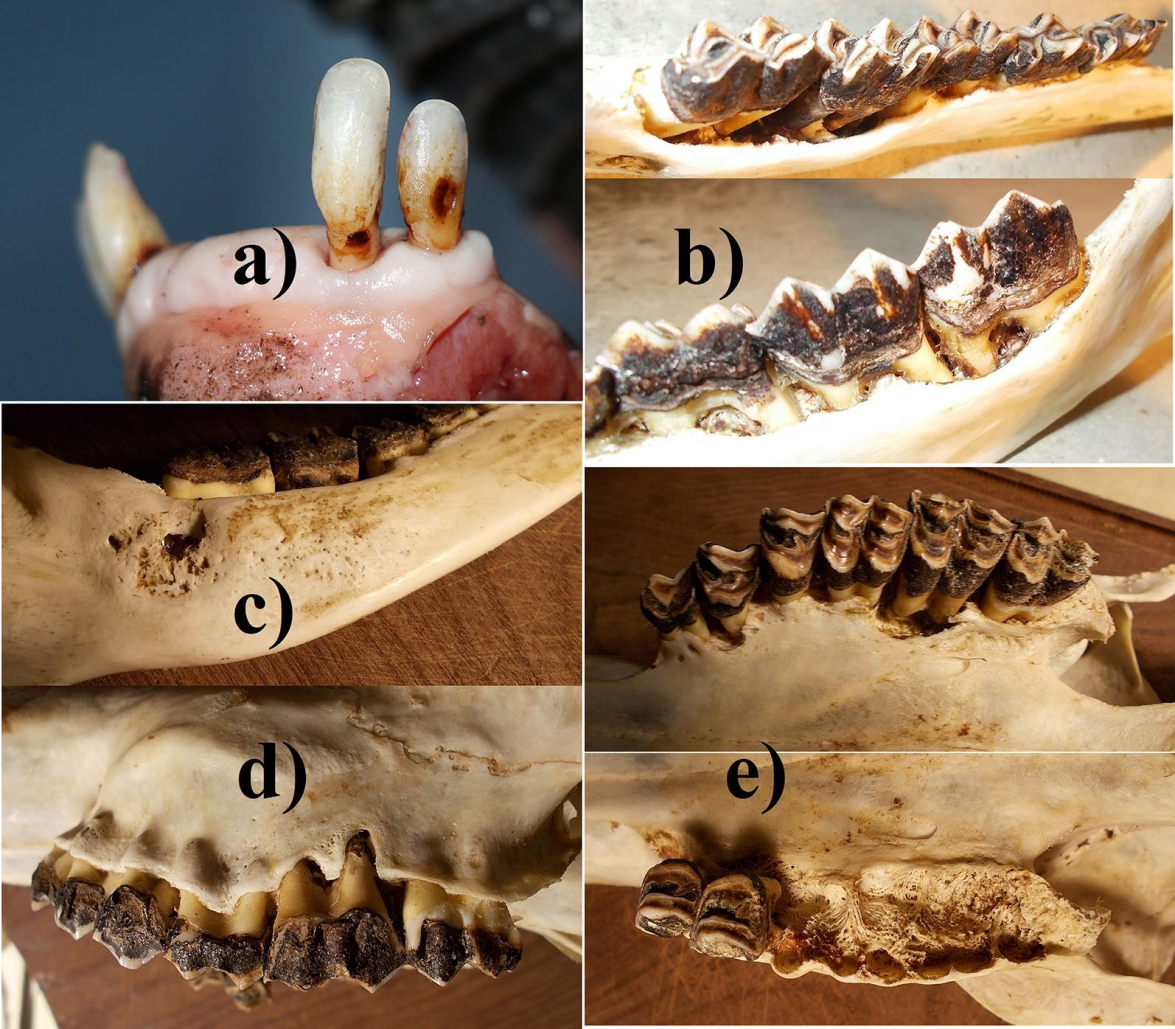
Case 6, female 2.5 years old: **a** loss of 4 central incisors with gums healed over, receded gum and exposed roots of remaining teeth; **b** extensive erosion of alveoli, exposed roots, reduced jaw height; **c** extensive perforation of the mandibular body, thickened mandibular body with osteoporotic spongy bone; **d** exposed maxillary roots; **e** erosions of the palate with porosification and perforations, extensive erosion of alveolar pockets

The fresh mandible showed 4 incisors missing (I1, I2), the gum completely healed but receded to expose roots of remaining teeth (Fig. 3a).

Several mandibular teeth revealed defective enamel, extensive erosion of several alveoli causing loose teeth, exposed roots, reduced jaw height, an extensive perforation of the left mandibular body (lingual), and probably secondary infections (Fig. 3b). That mandibular area had a thickened body that was transformed into osteoporotic spongy bone (Fig. 3c). The skull showed bone resorption from necrosis exposing roots of several maxillary molars and premolars, erosions of the palate resulting in porosification and even perforations, and extensive erosion of alveolar pockets (Fig. 3d, e). Without soft tissue, several teeth exfoliated. Some vertebras were asymmetrical.

#### Case 7

A female 3.5 years old had all front teeth, with gums covering all roots, and no clinical signs of disease. She was lactating and with a fawn.

### Discussion

#### Osteopathology

Clinical lesions were encountered in 6 of 7 huemul. One male was strongly limping while another male had a damaged hoof. Four animals lost 2–7 front teeth, with roots of remaining teeth exposed from gum recession: yet the oldest age was only 4–5 years. One animal had abnormally wide gaps between incisors and exposed roots from gum recession. A female with 4 lost front teeth and exposed roots exhibited severe bone necrosis, very loose teeth both in mandible and maxilla, and the mandibular body showing a massive perforation. These cases of acute periodontitis and parodontitis undoubtedly explain the poor level of fat deposits in the dead female, and advanced muscle atrophy in the male which had lost 7 frontal teeth. These lesions, their severity and prevalence, corroborate previous observations on 35 skeletons of Patagonian huemul deer [2]. The high prevalence of osteopathology in carcasses is now also substantiated for live huemul. The clinical severity of osteopathology in carcasses from Argentina (this study, 2) and Chile [6] demonstrate the severity of the disease status of these recently radio-collared huemul.

An important new discovery is that 57% of huemul had lost 2–7 front teeth in vivo even at young age, suggesting strong osteopathological processes resulting in age-independent premature exfoliation of teeth. Incisors were unmentioned in earlier analyses of carcasses [2], being commonly absent in decomposed jaws, since single conical roots have little hold in the alveoli.

Given the fresh dead huemul had not only lost incisors, but also exhibited severe mandibular and maxillary lesions, the other diseased but live individuals most certainly have similar additional lesions. These exfoliated front teeth resemble periodontosis (“broken-mouth”) described in sheep [11], except that symptoms in huemul also include lesions of premolars and molars, and affected young individuals. Observations of additional huemul at close distances revealed asymmetrical velvet growth and lumps beneath mandibles: therefore, likely other cases affected by clinical disease. Reduced foraging efficiency via exfoliated incisive teeth presents the most parsimonious explanation for observed impacts on body condition, low average age of 3.1 years [2], and recruitment rates too low to allow re-colonization of habitats used historically.

#### Etiological factors

In case of accompanying infections, it is fundamental to determine if they are of primary versus secondary origin. Fluorosis was discarded for Argentine huemul [6, 12]. However, iodine deficiency in southern Chile is documented in people [13] and livestock [14, 15]. In two Argentine provinces with huemul, goiter rates in 1965 among 20 year-old men were 33 and 48% [16]. Primary iodine deficiency thus may also affect huemul, but is aggravated by Se deficiency due to its key function in iodine metabolism. Se deficiency in huemul has been confirmed in southern Chile, with 73% deficient and 64% severely deficient [5], coinciding with Se deficient plants and livestock, including severe muscular dystrophy [5, 17, 18]. Soil Se levels (0.19 ppm) from areas used by extant huemul, shown to be at the *low end* of the range considered deficient, corroborate deficient levels in forage, livestock and huemul [19]. Moreover, the nexus between osteopathology in huemul and nutritional factors has been shown for a population in Chile: co-occurrent Se deficiency and severe degree of equivalent bone lesions [6].

Mammalian biochemistry (Se, iodine) allows direct extrapolations between species with high degrees of correlation [20], because Se functions at very basic biochemical levels including genetic coding: being the 21st naturally occurring amino acid [21]. Se deficiency not only reduces host defense, but also impairs bone metabolism, causing osteopenia and osteoarthritis [22, 23]. Similar environments elsewhere cause Se deficiency in ruminants, documented as underlying factor for periodontitis, mandibular thickening, premature tooth exfoliation, and reduced bone density [11, 24–26], similarly to lesions in huemul here [2] and from Chile [6]. Se is essential in thyroid metabolism, and thus plays a major role in iodine deficiency [6]. The combined known effects on bone metabolism present the most intelligible explanation for the osteopathology and lack of recovery. Importantly, Se and iodine deficiency also have a strong direct impact on reproduction, and thus on recruitment and potential to colonize new areas [17].

#### Aspect of nutritional ecology

Commonly, mammals have strong presence in good habitat (source areas), but they also occupy marginal areas, and even areas incapable of sustaining populations (sink areas). Most remaining huemul subpopulations (> 100) fail to recover, are diminishing, or have recently disappeared, indicating these habitats are not good enough as source areas [17]. Although environments with extant huemul contain highly heterogeneous landscapes due to topography, it remains a crucial fact that huemul are currently only using very restricted portions, which tend to be marginal or deficient regarding micronutrients. However, huemul formerly also utilized more fertile landscape portions, particularly during winter, namely places which became occupied with humans, their agriculture and livestock, or with towns and cities [17].

Several trace minerals like Se are consistently more concentrated in lower elevations in flood plains, valley bottoms, and in drier sites [27, 28]. Moreover, geological processes frequently result in mineral licks used by wild ruminants, and these occur most often, even exclusively, on winter ranges and at low elevations [29, 30]. Other wild ruminants make short trips during summer, to visit mineral licks every other months on winter ranges up to 2000 m lower in elevation, to replenish Se-deficient summer diets [31]. In the Columbian Andes, ruminants were Se deficient at high, but not at low elevation [32].

Yet the existing absence of recovery of huemul must not be an enigma. The nexus to nutritional ecology regarding huemul likely is their inaccessibility of most lowlands and traditional winter ranges, elimination of their migratory traditions, and concomitant elimination of source populations. Thus, remaining huemul populations are frequently restrained to marginal if not sink areas, or ecological traps. Moreover, subclinical effects may be the more frequent situation, yet these are best demonstrated mainly with production trials [33]. One remedy is the use of adaptive management by creating source population via breeding centers and reintroducing these huemul to formerly used habitat, which are considered to provide the necessary conditions to form source populations.

## Limitations

The presented data is limited by sample size and by itself does not allow generalizations outside of the study area.

## Additional files

**Additional file 1: Figure S1.** a) Patagonia; b) Study area: part of the Protected Park Shoonem used for marking huemul (red), containing lake la Plata centered at 44°51′S and 71°48′W.

**Additional file 2: Figure S2.** Snow condition at the lowest elevation of the region used by huemul. One of the females was marked with a radio collar shortly after.

## Abbreviation

Se: selenium
