# Supplementary figures and images for: Troubling disease syndrome in endangered live Patagonian huemul deer (Hippocamelus bisulcus) from the Protected Park Shoonem: unusually high prevalence of osteopathology
Source: BMC Res Notes. 2017 Dec 16;10:739. doi: 10.1186/s13104-017-3052-4 (PMC5732515; doi:10.1186/s13104-017-3052-4)

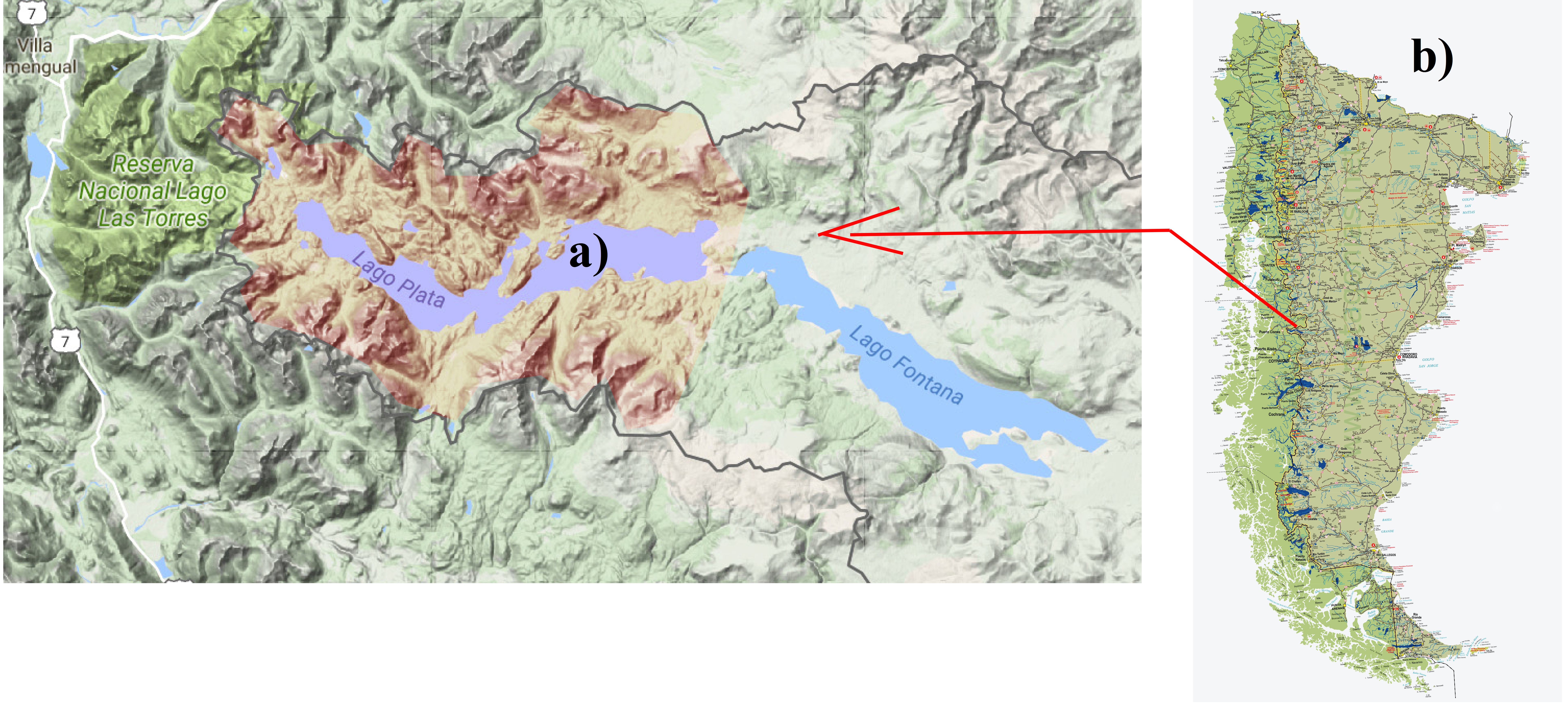

Supplement: Supplementary file 1 — Additional file 1: Figure S1. a) Patagonia; b) Study area: part of the Protected Park Shoonem used for marking huemul (red), containing lake la Plata centered at 44°51′S and 71°48′W. [file 13104_2017_3052_MOESM1_ESM.jpg]

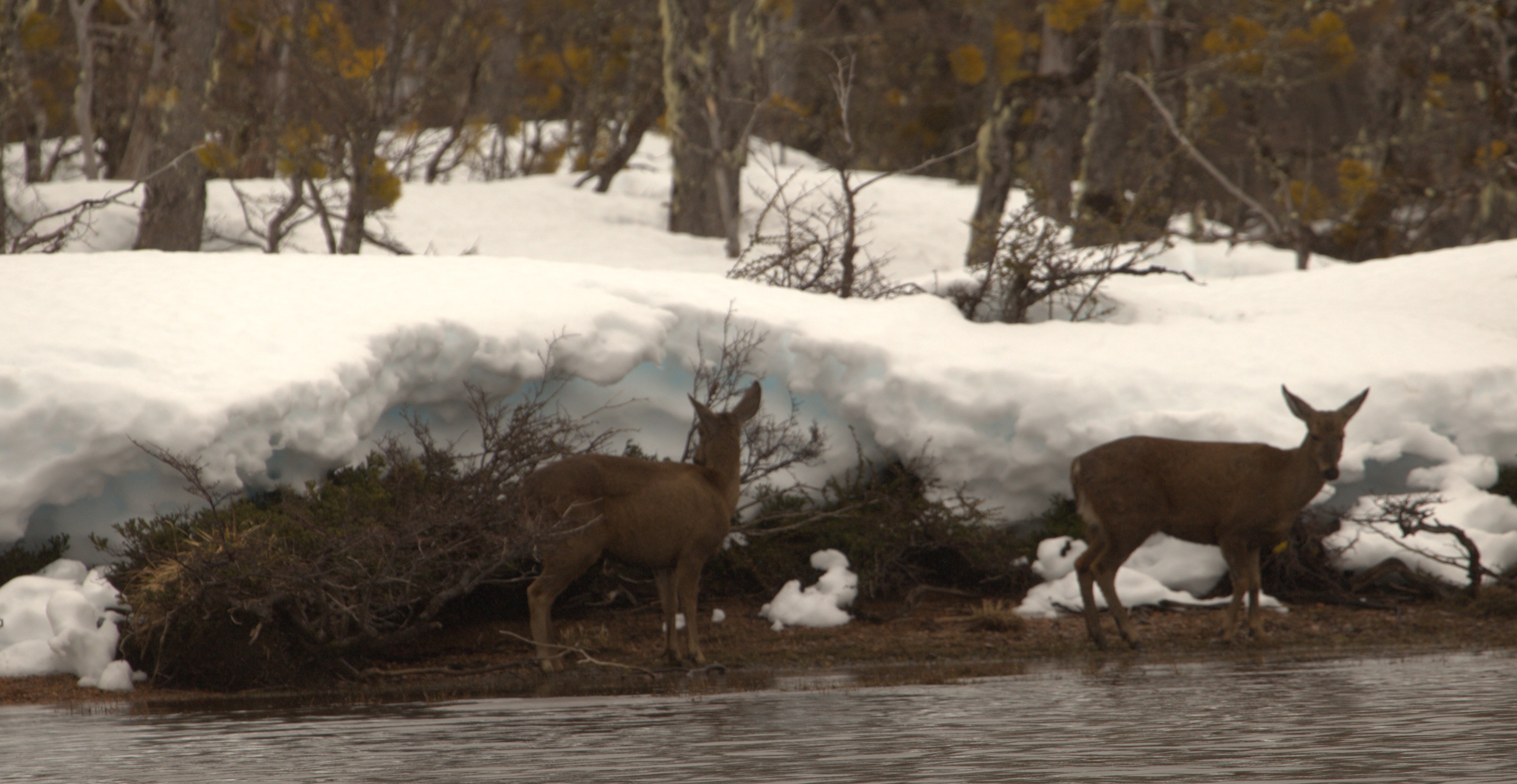

Supplement: Supplementary file 2 — Additional file 2: Figure S2. Snow condition at the lowest elevation of the region used by huemul. One of the females was marked with a radio collar shortly after. [file 13104_2017_3052_MOESM2_ESM.jpg]
